# Fatty Fish Intake Decreases Lipids Related to Inflammation and Insulin Signaling—A Lipidomics Approach

**DOI:** 10.1371/journal.pone.0005258

**Published:** 2009-04-23

**Authors:** Maria Lankinen, Ursula Schwab, Arja Erkkilä, Tuulikki Seppänen-Laakso, Marja-Leena Hannila, Hanna Mussalo, Seppo Lehto, Matti Uusitupa, Helena Gylling, Matej Orešič

**Affiliations:** 1 VTT Technical Research Centre of Finland, Espoo, Finland; 2 Department of Clinical Nutrition, School of Public Health and Clinical Nutrition, University of Kuopio, Kuopio, Finland; 3 Department of Internal Medicine, Kuopio University Hospital, Kuopio, Finland; 4 Department of Public Health, School of Public Health and Clinical Nutrition, University of Kuopio, Kuopio, Finland; 5 Information Technology Centre, University of Kuopio, Kuopio, Finland; 6 Department of Clinical Physiology and Nuclear Medicine, Kuopio University Hospital, Kuopio, Finland; National Institute of Child Health and Human Development/National Institutes of Health, United States of America

## Abstract

**Background:**

The evidence of the multiple beneficial health effects of fish consumption is strong, but physiological mechanisms behind these effects are not completely known. Little information is available on the effects of consumption of different type of fish. The aim of this study was to investigate how fatty fish or lean fish in a diet affect serum lipidomic profiles in subjects with coronary heart disease.

**Methodology and Principal Findings:**

A pilot study was designed which included altogether 33 subjects with myocardial infarction or unstable ischemic attack in an 8-week parallel controlled intervention. The subjects were randomized to either fatty fish (n = 11), lean fish (n = 12) or control (n = 10) groups. Subjects in the fish groups had 4 fish meals per week and subjects in the control group consumed lean beef, pork and chicken. A fish meal was allowed once a week maximum. Lipidomics analyses were performed using ultra performance liquid chromatography coupled to electrospray ionization mass spectrometry and gas chromatography. Multiple bioactive lipid species, including ceramides, lysophosphatidylcholines and diacylglycerols, decreased significantly in the fatty fish group, whereas in the lean fish group cholesterol esters and specific long-chain triacylglycerols increased significantly (False Discovery Rate *q*-value <0.05).

**Conclusions/Significance:**

The 8-week consumption of fatty fish decreased lipids which are potential mediators of lipid-induced insulin resistance and inflammation, and may be related to the protective effects of fatty fish on the progression of atherosclerotic vascular diseases or insulin resistance.

**Trial Registration:**

ClinicalTrials.gov NCT00720655

## Introduction

The evidence of the beneficial effects of consumption of fish and n-3 fatty acids on coronary heart disease (CHD) mortality as well as on cardiovascular risk factors is strong [1]–[3]. However, studies on the effects of different types of fish on CHD are limited. Controlled experimental studies have focused on the effects of supplemental n-3 long-chain polyunsaturated fatty acids (PUFA) rather than the effects of different types of fish consumption. Fatty acid content varies a lot between different fish species and depending on the preparation method. Fatty fish intake only is associated with increased serum concentration of n-3 long-chain PUFA [4], thus health benefits may vary depending on the type of fish meal consumed [5].

Epidemiological studies have shown associations between n-3 long-chain PUFA intake and insulin sensitivity [6], [7], but based on clinical trials, the evidence of positive effects on glucose metabolism is controversial [6], [8], [9], favoring no effect [10], [11]. However, n-3 PUFA could affect e.g. insulin receptor signaling [12], [13], inflammation [8], [14]–[15] or cell membrane fatty acid composition [13], [16], [17], which could further modulate insulin sensitivity. Multiple bioactive lipid components may play a role behind these mechanisms. Ceramides, for example, attenuate insulin signaling through multiple pathways [18]–[20], and diacylglycerols (DG) are identified as potential mediators of lipid-induced insulin resistance [18], [21]. Dietary exposure to eicosapentaenoic acid (EPA) and docosahexaenoic acid (DHA) is observed to reduce the production of DG and ceramides in murines [22].

Here we hypothesize that specific fish diet, rich in specific polyunsaturated fatty acids, affects not only the serum fatty acid composition but also the concentrations of bioactive lipids. The principal aim of the present study was to investigate how intakes of fatty fish or lean fish affect serum lipidomic profiles in subjects with CHD. We found that lipids related to impaired insulin signaling and inflammation decreased after an 8-week consumption of fatty fish, whereas this effect was not seen in the subjects who consumed lean fish or lean meat.

## Materials and Methods

The original protocol for this trial (in Finnish) and the CONSORT checklist are available as supporting information, Protocol S1 and Checklist S1.

### Subjects

**Figure 1:**
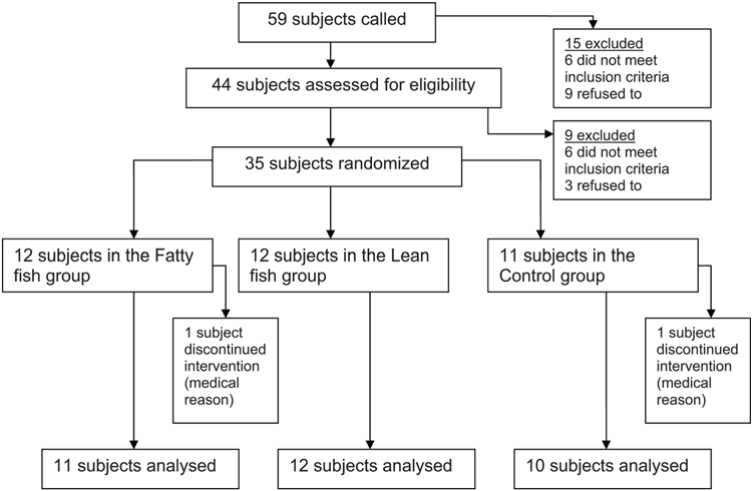
The CONSORT flow diagram.

Subjects and study design for this pilot study were described in detail by Erkkila et al [23]. In brief, altogether 44 subjects, identified from the discharge lists of the Kuopio University Hospital, were screened for the eligibility for the FISH -study, and 35 subjects were accepted (Figure 1). Thirty-tree of the subjects completed the study. All of them had acute myocardial infarction or unstable ischemic attack during the previous 3–36 months. To be included in the study, the subjects had to have age under 70 years, normal sinus rhythm, fasting serum triglyceride concentration ≤3.5 mmol/l, fasting serum cholesterol concentration ≤8 mmol/l, body mass index (BMI) 18.5–30 kg/m^2^, fasting plasma glucose concentration ≤7.0 mmol/l and they had to use betablockers. Subjects were excluded if they used other antiarrhythmic medications than betablockers or psychotropic drugs, were diagnosed with diabetes, atrial fibrillation, inflammatory bowel disease or abnormal liver, thyroid or kidney function or used excessive amounts of alcohol. Subjects who reported use of fish oil supplements or high fish intakes (>3 meals/week) during last three months were excluded from the study. All subjects were using statins in addition to betablockers, and 88% of subjects were using acetosalisylic acid, 45% ACE inhibitor, 39% oral anticoagulant, 27% calcium antagonist and 27% nitrate as well. Characteristics of the subjects in the intervention groups at screening visit have been published earlier [23].

The subjects gave written informed consent for the participation in the study. The study plan was approved by the Research Ethics Committee, Hospital District of Northern Savo.

### Study design

The study was an 8-week controlled, parallel intervention. The subjects were randomized to one of the following groups: fatty fish group (n = 11), lean fish group (n = 12) or control group (n = 10). Subjects in the fatty fish and lean fish groups were advised to consume fish 100–150 g/meal at least four times a week. Subjects in the fatty fish group consumed e.g. salmon, rainbow trout, Baltic herring, whitefish and vendace, and in the lean fish group e.g. pike, pike-perch, perch, saithe and cod. Subjects in the control group were instructed to consume less than 1 fish meal per week and to eat meals made with lean meat (beef or pork) or chicken without skin. Otherwise all subjects were instructed by a clinical nutritionist to follow a diet recommended for CHD patients [24]. They were advised to avoid sources of saturated fat like cream and butter in food preparation. Subjects continued to use the medications prescribed by their physicians.

Measurements were performed at baseline (week 0) and week 8. Height, weight and blood pressure were measured and blood samples were drawn after a 12-h overnight fast. Blood samples drawn at baseline and week 8 were used for lipidomic and fatty acid analyses.

### Dietary intake

Subjects' habitual dietary intake was estimated by a 4-day food record (consecutive days, 3 weekdays and one weekend day) at the baseline. Dietary compliance was monitored by using a 7-day food record kept twice during the intervention (weeks 3 and 7) and with the daily record of fish consumption. Nutrient intake was calculated using the Micro-Nutrica® dietary analysis program (version 2.5, Finnish Social Insurance Institute, Turku, Finland).

### Biochemical analyses

Blood samples were drawn from an antecubital vein. Concentrations of serum total, LDL and HDL cholesterol and triglycerides (TG) were analyzed using commercial kits (981813, 981656, 981655 and 981786, respectively, Thermo Electron Corporation, Vantaa, Finland) and Thermo Fisher Konelab 20XTi Analyzer (Thermo Electron Corporation, Vantaa, Finland). Serum insulin concentration was analyzed with a chemiluminescent immunoassay (ACS 180 Plus Automated Chemiluminescence System, Bayer Diagnostics, USA). Plasma glucose was analyzed by using Thermo Fischer Konelab 20XTi Analyzer (Thermo Electron Corporation, Vantaa, Finland) and kit 981779 (Thermo Electron Corporation, Vantaa).

### Fatty Acid Analyses

The fatty acid method was used to measure esterified fatty acids. The coefficient of variation of the reported method is <1% for each reported fatty acid.

#### Sample preparation

Lipids from plasma samples (50 µl) were extracted by a mixture of chloroform and methanol (2∶1, 100 µl) after addition of internal standard (20 µl, heptadecantrienoate TG C17:0 500 mg/l+FFA C17:0 500 mg/l). The lower layer was separated and evaporated into dryness under nitrogen flow. The residue was dissolved into petroleumether (700 µl, boiling point 40–60°C). Bound fatty acids were transmethylated with NaOME (250 µl, sodium methoxide in dry methanol 0,5 M) by boiling at 45°C for 5 minutes. The mixture was acidified by adding 15% solution of NaHSO_4_ (500 µl). The fatty acids methyl esters (FAME) and free fatty acids (FFA) were extracted by petroleum ether (300 µl). The petroleumether layer containing FAME and FFA was separated into a glass vial, evaporated under nitrogen flow and redissolved into hexane (50 µl) and transferred into a glass microinsert for GC analysis.

#### Chromatography

2 µl aliquots were used for GC injection at 260°C. The split ratio was 1∶13 and the injector liner was deactivated with glass wool. The Agilent 5890 series II Gas Chromatography equipped with a 25 meter HP-FFAP column (diameter 0.32 mm) was used. Helium was used as carrier gas at a total flow of 30 ml/min. The initial oven temperature was 70°C and the temperature was increased at rate of 7°C/min until 240°C. The fatty acids were detected by flame ionization detector at 300°C.

### Lipidomic analyses

Lipidomics analyses were performed as described earlier [25]. In brief, plasma samples (10 µl) were diluted with 10 µl sodium chloride (0,9%) and 20 µl of an internal standard mixture containing 10 lipid classes. The lipids were extracted with chloroform/methanol (2∶1, 100 µl). An external standard mixture containing 3 labeled standards (10 µl) was added to the lipid extracts. Half of the lipid extract was transferred into another HPLC vial as a replicate. The sample order for LC/MS analysis was determined by randomization.

#### Ultra performance liquid chromatography coupled to electrospray ionization mass spectrometry (UPLC-ESI-MS)

Lipid extracts were analyzed on a Waters Q-Tof Premier mass spectrometer combined with an Acquity Ultra Performance Liquid chromatography. The column was an Acquity UPLC™BEH C18 1×50 mm with 1.7 particles. The binary solvent system includes A. water (1% 1 M NH_4_Ac, 0.1% HCOOH) and B. isopropanol:acetonitrile (1∶2.5, 1% 1 M NH_4_Ac, 0,1% HCOOH). The gradient started from 65% A/35% B and reached 100% B in 6 minutes and remains at this level for next 7 minutes. The flow rate was 200 µl/min and the injection volume was 1 µl. The profiling of lipid extracts was carried out on Waters Q-Tof premier mass spectrometer using ESI+ mode. The voltages of the sampling cone and capillary were 45.0 V and 2.9 kV, respectively. The source temperature was set at 120°C and nitrogen was used as desolvation gas (795 L/h) at 270°C. The data were collected in centroid mode at mass range of m/z 300–1200 with a scan time of 0.20 sec. Reserpine (50 µl/L) was used as the lock spray reference compound at a flow rate of 10 µl/min and the scan was done at 10 s frequency.

#### Data processing

Data processing was performed using MZmine software version 0.60 [26]. MZmine processing consisted of four phases: 1) peaks detection, 2) aligning based on retention times and m/z values, 3) filtering out peaks appearing only few samples and 4) gap filling. The quality of results was controlled by checking that standard peaks were found. The normalization of lipidomics data were performed as follows: all monoacyl lipids except cholesterol esters, such as monoacyl-glycerophospholipids, were normalized with lysophosphatidylcholine PC(17:0/0:0) internal standard, all diacyl phosphocholines and SMs were normalized with phosphatidylcholine PC(17:0/17:0), the diacyl ethanolamine phospholipids were normalized with phosphatidylethanolamine PE(17:0/17:0), the diacyl phosphoserine phospholipids were normalized with phosphatidylserine PS(17:0/17:0), the diacyl phosphates and phosphoglycerols were normalized with phosphatidic acid PA(17:0/17:0), and the triacylglycerols and cholesterol esters with triacylglycerol TG(17:0/17:0/17:0). Unidentified species were normalized by PC(17:0/0:0) for retention time <300 seconds, PC(17:0/17:0) for the retention time between 300 s and 410 s, and TG(17:0/17:0/17:0) for higher retention times.

### Statistical analyses

Biochemical data were statistically analyzed using the SPSS statistical software (version 14.0, SPSS Inc., Chicago, IL) and R software version 2.4.1 [27]. The data are expressed as mean±SD. Fold changes for the lipids were calculated dividing the endpoint values by the baseline values. The HOMA insulin resistance index (HOMA-IR) was calculated as following: fP-Insulin (mU/l)×fP-Glucose (mmol/l)/22.5. The normality of distributions of the variables was tested with the Kolmogorov-Smirnov test with Lilliefors significance correction. The variables with abnormal distribution were normalized with logarithmic transformation or a non-parametric test was used if normal distribution was not achieved with transformation. Paired samples *t*-test or Wilcoxon signed ranks test was used when comparing baseline and endpoint values within the groups. Spearman rank correlation was used to calculate correlation coefficients. *P*-values <0.05 were considered significant. For the high-dimensional lipidomics dataset, the *P*-values were corrected for multiple comparisons by calculating the False Discovery Rate (FDR) *q*-values [28]. In order to compare the group differences at baseline and after the intervention, one-way analysis of variance (ANOVA) was performed on lipidomics data for each lipid separately at baseline and after the intervention. The FDR *q*-values were also calculated from the ANOVA *P*-values. *q*-values <0.05 were considered significant.

Mixed model was used to assess relation between lipidomics variables and sum of EPA and DHA. Due to the skewness of the variables (both dependent variables and sum of DHA and EPA) they were transformed to logarithmic scale. And after that they were standardized (to have zero mean and standard deviation of 1) in order to get comparable (or standardized) regression coefficients of mean variable for dependent variables. Every transformed lipidomics variable was modeled by an own model with transformed mean variable and time as a fixed and person as a random explanatory variables. In order to correct for multiple comparisons, FDR *q*-values are reported in addition to *P*-values.

## Results

### Clinical characteristics and dietary intake

Clinical characteristics at baseline and after the intervention are presented in Table 1. There were no significant changes in the fatty or lean fish groups. In the control group, serum total and HDL cholesterol concentrations decreased (*P* = 0.027 and 0.028, respectively) during the intervention. The use of medications did not differ among the groups [23].

**Table 1:** Clinical characteristics at baseline and after the 8-week intervention (mean±SD).

	Fatty fish (n = 11)			Lean fish (n = 12)			Control (n = 10)		
	0 wk	8 wk	*P*-value	0 wk	8 wk	*P*-value	0 wk	8 wk	*P*-value
Body mass index (kg/m2)	26.7±3.1	26.8±3.2	0.602†	27.8±2.1	27.5±1.9	0.051	27.0±2.9	26.9±2.9	0.190
Serum cholesterol (mmol/l)	3.8±0.6	3.5±0.6	0.361	3.7±0.7	3.7±0.6	0.805	4.7±0.8	4.3±0.8	0.027
LDL cholesterol (mmol/l)	1.9±0.3	1.9±0.3	0.585	2.0±0.6	2.0±0.5	0.975	2.6±0.6	2.5±0.6	0.162
HDL cholesterol (mmol/l)	1.4±0.4	1.5±0.4	0.246†	1.3±0.3	1.3±0.3	0.568	1.4±0.5	1.3±0.4	0.028
Serum triacylglycerols (mmol/l)	1.3±0.7	1.1±0.4	0.278	1.1±0.6	1.1±0.6	0.981†	1.9±1.2	1.7±0.5	0.403
Plasma glucose (mmol/l)	6.1±0.9	6.1±0.5	1	5.6±0.4	5.5±0.4	0.234‡	5.7±0.3	5.6±0.3	0.185
Serum insulin (mU/l)	13.8±12.4	12.3±8.8	0.425†	9.7±5.1	8.0±3.8	0.175	12.6±7.3	11.0±4.7	0.392
HOMA-IR§	4.1±4.8	3.4±2.8	0.547†	2.4±1.2	2.0±1.0	0.155	3.2±1.9	2.7±1.2	0.41†

* Paired-samples *t*-test.

† Logarithmic values used in statistical analyses due to the abnormal distribution.

‡ Wilcoxon signed ranks test used due to the abnormal distribution even after logarithmic transformation.

§ HOMA-IR, HOMA insulin resistance index.

Self-reported compliance with the diet was good. Average fish consumption during the study was 4.3, 4.7 and 0.6 fish meals per week in the fatty fish, lean fish and control groups, respectively. Nutrient intake at baseline and during the study was reported earlier in detail [23]. In the fatty fish group the dietary intake of total PUFA, EPA, DHA and α-linolenic acid increased (Table 2). In the lean fish group, the intakes of total energy, total fat as well as SAFA, PUFA and MUFA decreased. In the control group, the intake of EPA decreased during the intervention.

**Table 2:** Daily dietary intakes at baseline and during the study (mean±SD).

	Fatty fish (n = 11)			Lean fish (n = 12)			Control (n = 10)		
	baseline	during the study	*P*-value*	baseline	during the study	*P*-value*	baseline	during the study	*P*-value*
Energy (kJ)	6179±1271	6586±1151	0.081	7286±1902	6738±1803	0.038	8251±3052	7273±1784	0.161
Total fat (E%)	31±6	31±5	0.992	31±4	27±4	0.003	31±7	29±5	0.363
SAFA (E%)	11±4	9±3	0.321	10±3	8±2	0.004†	12±3	10±3	0.108
MUFA (E%)	11±3	10±2	0.588†	10±1	9±2	0.027	10±2	10±2	0.766
PUFA (E%)	6±1	7±1	0.016†	7±1	6±1	0.712	5±2	6±1	0.442†
EPA (g)	0.13±0.07	0.30±0.12	<0.001	0.15±0.18	0.12±0.05	0.577†	0.11±0.11	0.05±0.03	0.035
DHA (g)	0.31±0.19	0.77±0.29	0.001	0.36±0.54	0.32±0.10	0.219†	0.24±0.25	0.11±0.08	0.077
α-linolenic acid (g)	1.4±0.7	1.9±0.8	0.028†	1.7±0.8	1.6±0.6	0.49	1.4±0.8	1.5±0.7	0.421†
Linoleic acid (g)	6.5±2.2	8.3±1.9	0.065	8.0±3.6	7.5±3.0	0.40	9.07±6.8	7.4±3.6	0.602†

Values at baseline are based on a 4-day food records, and values during the study are based on a 7-day food records kept twice during the study.

* Paired-samples *t*-test.

† Logarithmic values used in statistical analyses due to the abnormal distribution.

### Lipidomics

Proportions of plasma oleic acid (18:1n-9) and dihomo-γ-linolenic acid (20:3n-6) decreased and those of α-linolenic (18:3n-3), arachidonic (20:4n-6), EPA (20:5n-3), docosapentaenoic (22:5n-3) and DHA (22:6n-3) increased in the fatty fish group (Table 3). In the lean fish group, only the proportion of cis-vaccenic acid (18:1n-7) increased. No significant changes in plasma fatty acids were observed in the control group.

**Table 3:** Plasma fatty acids (%) at baseline and after the 8-week intervention (mean±SD).

		Fatty fish (n = 11)			Lean fish (n = 12)			Control (n = 10)		
		0 wk	8 wk	*P*-value	0 wk	8 wk	*P*-value	0 wk	8 wk	*P*-value
Myristic acid	(14:0)	0.73±0.41	0.72±0.32	0.996†	0.65±0.41	0.63±0.26	0.71†	0.92±0.40	0.83±0.28	0.30
Palmitic acid	(16:0)	24.46±2.29	23.73±1.78	0.250	24.36±1.95	24.00±1.26	0.51	24.84±1.85	24.11±1.70	0.11
Palmitoleic acid	(16:1n-7)	2.28±1.14	2.06±0.75	0.270	2.17±0.63	2.10±0.69	0.69	2.67±1.11	2.63±0.91	0.83
Stearic acid	(18:0)	8.33±0.99	8.51±1.07	0.390	8.14±0.95	8.14±1.08	0.98	7.76±0.96	7.45±0.73	0.31
Oleic acid	(18:1n-9)	22.68±3.72	20.11±3.64	0.009	21.61±2.61	22.24±3.25	0.50	26.22±5.05	26.64±2.87	0.70
Cis-vaccenic acid	(18:1n-7)	2.38±0.32	2.36±0.33	0.680	2.38±0.30	2.48±0.37	0.04	2.62±0.46	2.68±0.50	0.54
Linoleic acid	(18:2n-6)	19.85±3.55	19.92±1.96	0.920	21.16±1.49	21.10±2.75	0.94	18.73±4.53	19.78±4.89	0.14
γ-linolenic acid	(18:3n-6)	0.60±0.32	0.63±0.34	0.82†	0.52±0.32	0.48±0.21	0.57	0.46±0.19	0.52±0.19	0.28
α-linolenic acid	(18:3n-3)	1.03±0.40	1.20±0.41	<0.001†	1.04±0.31	1.07±0.29	0.55	1.08±0.37	1.21±0.37	0.18
Di-homo-γ-linolenic acid	(20:3n-6)	1.92±0.42	1.62±0.39	0.005	1.82±0.34	1.85±0.33	0.74	1.63±0.35	1.67±0.27	0.73
Arachidonic acid	(20:4n-6)	7.81±1.73	7.18±1.61	0.140	8.08±1.45	8.14±1.70	0.64‡	6.88±1.50	7.04±0.85	0.68
Eicosapentaenoic acid	(20:5n-3)	2.41±0.71	4.65±2.02	<0.001†	2.52±1.11	2.15±1.10	0.27†	1.98±1.38	1.54±0.58	0.37†
Docosapentaenoic acid	(22:5n-3)	0.97±0.15	1.14±0.17	0.016	1.02±0.23	0.96±0.31	0.07†	0.87±0.21	0.85±0.17	0.77
Docosahexaenoic acid	(22:6n-3)	4.53±0.80	6.15±1.17	0.001	4.53±1.47	4.69±1.86	0.72	3.34±1.67	3.03±0.80	0.43

* Paired samples *t*-test.

† Logarithmic values used in statistical analyses due to the abnormal distribution.

‡ Wilcoxon signed ranks test used because the distribution of variance was not normal even after logarithmic transformation.

**Figure 2:**
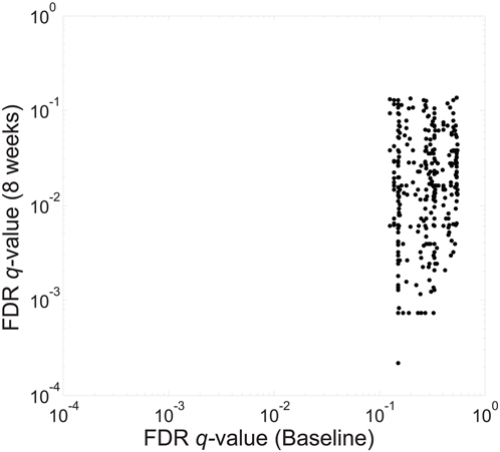
False Discovery Rate *q*-values based on one-way ANOVA test for each of the 307 identified lipids (represented by black dots) from the lipidomics dataset across the three intervention groups at baseline (horizontal axis) and after the 8 week intervention (vertical axis). None of the lipids reached *q*<0.05 at baseline.

**Figure 3:**
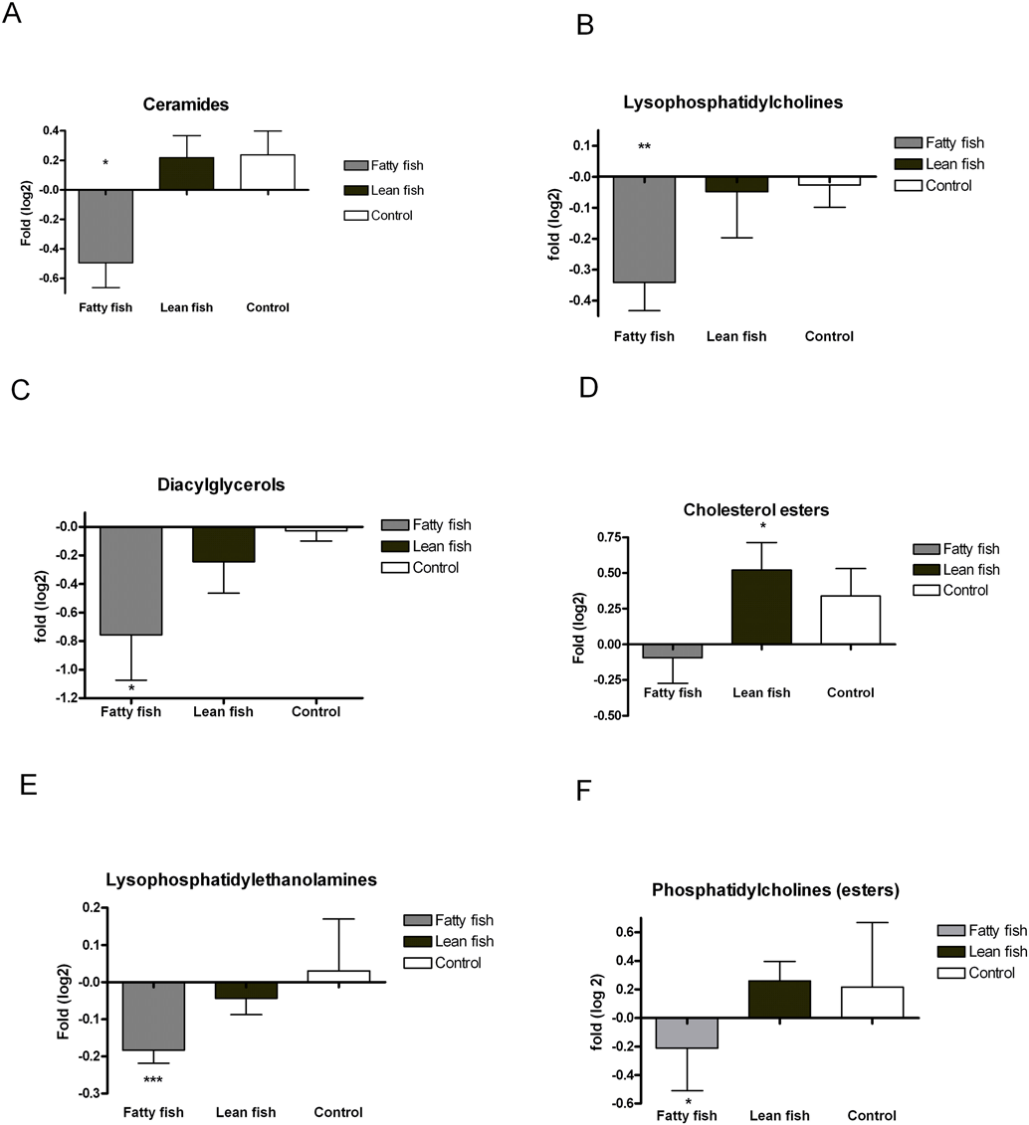
Bar charts of fold changes for different lipid classes (calculated as a sum of lipid concentrations within a class) in each group. *, *P*<0.05; **, *P*<0.01; ***, *P*<0.001. P-values were calculated using paired samples *t*-test (differences inside the groups before and after intervention).

A total of 307 lipids were identified and quantified by the UPLC/MS lipidomics platform. We first established that there were no significant lipid changes between the groups at baseline (Figure 2). A total of 240 lipids were different across the three groups after the intervention at FDR q<0.05 (Figure 2). When comparing within-person changes across the three intervention groups, no lipids were significantly altered in the control group, while 63 and 5 lipids changed in the fatty and lean fish groups, respectively (Table 4). Multiple bioactive lipid species, including ceramides, lysophosphatidylcholines (lysoPC), DGs, phosphatidylcholines and lysophosphatidylethanolamines, decreased significantly in the fatty fish group, whereas in the lean fish group cholesterol esters and specific long-chain triacylglycerols increased significantly (Table 4 and Figure 3).

**Table 4:** Main lipidomic changes across the three groups.

LipidName	*q*-value*	*q*-value*	*P* † (8 wk)	*P* † (8 wk)	*P* † (8 wk)	Within-person	*q*-value‡	Within-person	*q*-value‡
	0 wk	8 wk	Fatty fish *vs*. Control	Lean fish *vs*. Control	Fatty *vs*. Lean fish	log2 Fold Fatty fish	Fatty fish	log2 Fold Lean fish	Lean fish
**Cer(d18:1/23:0)**	0.32	0.0163	0.0060	0.3629	0.1438	**0.72**	**0.0316**	1.14	0.3434
**Cer(d18:1/24:1)**	0.15	0.0112	0.0100	0.1179	0.2048	**0.79**	**0.0418**	1.30	0.1861
**ChoE(20:5)**	0.15	0.0014	0.0069	0.7417	0.0022	**2.48**	**0.0145**	**1.53**	**0.0450**
**DG(32:5)**	0.33	0.0116	0.0126	0.2925	0.0406	**0.70**	**0.0350**	0.93	0.2367
**DG(40:1)**	0.41	0.0163	0.0242	0.9314	0.0278	**0.71**	**0.0431**	0.96	0.2776
**DG(44:7)**	0.33	0.0231	0.0464	0.7112	0.0488	**0.69**	**0.0340**	1.00	0.3416
**PC(32:0)**	0.53	0.0092	0.0112	0.4808	0.0183	**0.81**	**0.0431**	1.11	0.4004
**PC(32:3)**	0.34	0.0137	0.0165	0.9481	0.0244	**0.64**	**0.0046**	1.10	0.4318
**PC(34:1)**	0.18	0.0016	0.0009	0.0135	0.0937	**0.80**	**0.0340**	1.27	0.2251
**PC(34:2)**	0.25	0.0074	0.0060	0.1091	0.1285	**0.80**	**0.0340**	1.21	0.2387
**PC(34:3)**	0.52	0.0157	0.0360	0.8519	0.0094	**0.60**	**0.0346**	2.30	0.3328
**PC(36:0)**	0.30	0.0194	0.0323	0.3009	0.1201	**0.76**	**0.0219**	1.00	0.3821
**PC(36:2)**	0.18	0.0131	0.0283	0.0766	0.3837	**0.83**	**0.0285**	1.28	0.1559
**PC(36:4)**	0.15	0.0026	0.0007	0.0694	0.0466	**0.75**	**0.0145**	1.31	0.1179
**PC(36:5)**	0.27	0.0007	0.0031	0.7644	0.0005	**1.59**	**0.0219**	1.18	0.2147
**PC(38:4)**	0.14	0.0148	0.0201	0.1403	0.2482	**0.76**	**0.0144**	1.34	0.1387
**PC(38:6)**	0.33	0.0007	0.0000	0.0147	0.0272	**0.78**	**0.0219**	1.34	0.2222
**PC(38:8)**	0.24	0.0227	0.0289	0.8910	0.0598	**0.73**	**0.0215**	1.01	0.3821
**PC(40:6)**	0.20	0.0061	0.0017	0.0671	0.1738	**0.79**	**0.0092**	1.47	0.1324
**PC(32:0e)**	0.15	0.0299	0.0619	0.2177	0.4420	**0.85**	**0.0405**	1.11	0.2511
**PC(32:1e)**	0.15	0.0282	0.1302	0.0546	0.9694	**0.80**	**0.0260**	1.08	0.3434
**PC(34:0e)**	0.43	0.0074	0.0055	0.2738	0.0460	**0.74**	**0.0340**	0.99	0.3434
**PC(34:3e)**	0.30	0.0502	0.1275	0.4267	0.3502	**0.87**	**0.0470**	1.25	0.1731
**PC(36:5e)**	0.29	0.0168	0.0301	0.2182	0.1671	**0.81**	**0.0431**	1.19	0.2519
**PC(36:5e)**	0.29	0.0215	0.0333	0.2221	0.2432	**0.82**	**0.0431**	1.15	0.3416
**PC(38:5e)**	0.28	0.0073	0.0075	0.0464	0.2917	**0.80**	**0.0418**	1.19	0.3328
**PC(38:6e)**	0.27	0.0292	0.0437	0.6380	0.1409	**0.75**	**0.0418**	1.05	0.4004
**PC(40:4e)**	0.15	0.0519	0.0867	0.2897	0.6964	**0.80**	**0.0340**	1.05	0.4137
**PE(32:1)**	0.33	0.0225	0.0409	0.5987	0.0428	**0.73**	**0.0336**	1.11	0.4004
**PE(34:3e)**	0.45	0.0049	0.0027	0.1297	0.0424	**0.80**	**0.0442**	1.15	0.3699
**PE(36:2e)**	0.48	0.0030	0.0021	0.0576	0.0407	**0.69**	**0.0260**	1.07	0.4004
**PE(36:5e)**	0.52	0.0040	0.0051	0.1544	0.0144	**0.85**	**0.0418**	1.06	0.3254
**PE(36:6e)**	0.33	0.0028	0.0358	0.2720	0.0006	**1.29**	**0.0340**	1.10	0.4004
**PE(38:5e)**	0.33	0.0013	0.0004	0.0482	0.0088	**0.80**	**0.0431**	1.25	0.1221
**PS(32:0)**	0.42	0.0148	0.0254	0.6437	0.0202	**0.74**	**0.0486**	1.21	0.4191
**lysoPC(16:0)**	0.31	0.0167	0.0620	0.0413	0.8165	**0.86**	**0.0219**	1.07	0.4321
**lysoPC(16:1)**	0.35	0.0226	0.0665	0.0808	0.6391	**0.80**	**0.0219**	1.03	0.3821
**lysoPC(18:0)**	0.28	0.0416	0.2450	0.1176	0.6576	**0.84**	**0.0291**	1.11	0.4237
**lysoPC(18:0e)**	0.33	0.0217	0.0333	0.7159	0.0736	**0.70**	**0.0291**	1.00	0.3821
**lysoPC(18:1)**	0.34	0.0148	0.0318	0.0458	0.6183	**0.78**	**0.0260**	1.04	0.4004
**lysoPC(18:1e)**	0.47	0.0176	0.0435	0.0728	0.5809	**0.75**	**0.0046**	0.87	0.1308
**lysoPC(18:2)**	0.48	0.0585	0.1557	0.5233	0.3675	**0.80**	**0.0316**	1.01	0.3821
**lysoPC(18:2e)**	0.32	0.0384	0.1048	0.2481	0.5073	**0.79**	**0.0260**	1.09	0.4364
**lysoPC(20:3)**	0.25	0.0028	0.0005	0.1476	0.0331	**0.69**	**0.0219**	1.11	0.3434
**lysoPC(20:4)**	0.33	0.0316	0.0502	0.3135	0.3082	**0.73**	**0.0219**	1.03	0.4004
**lysoPE(19:2e)**	0.40	0.0157	0.0366	0.0576	0.6570	**0.85**	**0.0219**	1.03	0.4042
**lysoPE(20:0e)**	0.28	0.0058	0.0038	0.1484	0.0429	**0.60**	**0.0101**	0.90	0.2045
**TG(51:2)**	0.41	0.0148	0.0021	0.1537	0.3702	**0.72**	**0.0409**	1.29	0.4067
**TG(52:2)**	0.15	0.0007	0.0004	0.0021	0.3932	**0.77**	**0.0489**	2.08	0.1279
**TG(53:2)**	0.15	0.0008	0.0002	0.0028	0.3282	**0.74**	**0.0431**	1.57	0.2511
**TG(54:1)**	0.45	0.0083	0.0086	0.1742	0.0464	**0.72**	**0.0431**	1.14	0.3989
**TG(54:2)**	0.23	0.0007	0.0004	0.0013	0.4665	**0.73**	**0.0431**	1.74	0.1861
**TG(55:3)**	0.29	0.0023	0.0003	0.0139	0.2370	**0.75**	**0.0486**	1.35	0.3821
**TG(56:4)**	0.15	0.0042	0.0013	0.0739	0.1085	**0.72**	**0.0418**	1.71	0.1869
**TG(56:5)**	0.15	0.0007	0.0002	0.0004	0.5019	**0.74**	**0.0405**	1.88	0.1005
**TG(56:7)**	0.15	0.0327	0.3281	0.3476	0.0662	1.63	0.1218	**1.97**	**0.0450**
**TG(56:8)**	0.15	0.0062	0.0282	0.9410	0.0180	2.24	0.0662	**1.82**	**0.0439**
**TG(58:8)**	0.14	0.0163	0.1217	0.5548	0.0275	1.60	0.1155	**1.98**	**0.0439**
**TG(58:9)**	0.15	0.0058	0.0180	0.8328	0.0151	1.86	0.0763	**1.75**	**0.0481**

Since 240 lipids reached *q*<0.05 at 8 weeks based on one-way ANOVA across the three groups, additional criteria were applied to limit the number of lipids for clarity. Only lipids with within-person changes in Lean or Fatty fish group significant at *q*<0.05 are included. No lipids were changed significantly within the Control group. For isobaric lipid species of the same class, total number of carbons and double bonds, only one specie with the highest mean concentration is listed.

* FDR *q*-values calculated from *P*-value distribution obtained from one-way ANOVA.

† Two-sided *t*-test.

‡ FDR *q*-values calculated from *P*-value distribution obtained from paired samples *t*-test.

The mixed model revealed that the plasma contents of EPA and DHA were strongly correlated with the plasma long chain TG, cholesterol ester and lysoPC with EPA bound (ChoE(20:5) and lysoPC(20:5)), and with specific phosphatidylcholines (Table 5). They were also significantly related with the ether bonded phosphatidylethanolamines (plasmalogens) (Table 5).

**Table 5:** Lipids related with plasma content of sum of EPA and DHA based on mixed model.

Lipids	Coefficient	Standard error	*P*-value	FDR *q*-value
ChoE(20:5)*	0.52	0.09	0.001	0.001
lysoPC(20:5)†	0.58	0.11	0.001	0.001
PC(36:5)‡	0.56	0.10	0.001	0.001
PC(38:5)	0.38	0.12	0.003	0.026
PC(38:6e)	0.34	0.12	0.009	0.068
TG(60:11)§	0.46	0.12	0.001	0.009
TG(58:9)	0.42	0.11	0.001	0.013
TG(58:8)	0.42	0.11	0.001	0.013
TG(58:10)	0.42	0.12	0.001	0.016
TG(57:8)	−0.41	0.12	0.001	0.017
TG(56:8)	0.39	0.11	0.001	0.017
TG(54:1)	−0.39	0.12	0.002	0.026
TG(59:13)	0.37	0.12	0.003	0.026
TG(58:8)	0.37	0.12	0.004	0.032
TG(52:0)	−0.36	0.12	0.004	0.032
TG(40:3)	−0.34	0.12	0.007	0.056
TG(51:2)	−0.34	0.12	0.008	0.056
TG(56:9)	0.34	0.12	0.009	0.056
PE (38:5e)^a^	−0.33	0.13	0.014	0.067
PE (36:6e)	0.31	0.13	0.020	0.068
PE(36:2e)	−0.26	0.12	0.037	0.074
PE(42:1e)	0.27	0.13	0.044	0.076

Lipids with *P*-value <0.001 and phosphatidylethanolamines (plasmalogens) with *P*-value <0.05 reported.

* ChoE, Cholesterol ester.

† lysoPC, lysophosphatidylcholine.

‡ PC, phosphatidylcholine.

§ TG, triacylglycerol.

a PE, phosphatidylethanolamine.

## Discussion

We investigated the effect of fatty fish, lean fish or lean meat intake on plasma lipidomic profile in individuals with CHD. We found that the 8-week consumption of fatty fish altered the lipidomic profile markedly, while the lean fish or control diet did not cause such changes. Interestingly, the serum lipids found affected by the fatty fish diet are associated with insulin signaling and inflammation [19]–[21], [29], [30].

Fatty acid analysis revealed the increases in plasma content of all long chain n-3 fatty acids in the fatty fish group, which confirms the good compliance to the diet. The subjects were instructed to use low erucic acid rapeseed oil based liquid margarines or vegetable oils (rapeseed oil most commonly used in Finland) for frying the fish, which increased the intake of ALA during the study.

Based on food records, the total energy intake decreased significantly in the lean fish group. However, there were no significant changes in their weight or body mass index. In the control group, the intake of EPA decreased during the intervention, but no changes in plasma fatty acids were seen in this group. The fish consumption was restricted to one fish meal per week in the control group, which was less than subjects habitually consumed. Seventy % of the control subjects consumed habitually 1–2 fish meals per week and 30% consumed less than one fish meal per week.

The principal findings of the present study, as revealed by the lipidomic analysis, were diminished DGs, ceramides and lysoPCs in the fatty fish group (Table 4 and Figure 3). DG is the potential mediator of lipid-induced insulin resistance via activation of novel protein kinase C (nPKC), which inhibits insulin action and is also related to inflammatory response [18], [21]. Ceramides are suggested to attenuate insulin signaling through multiple pathways, but the exact mechanism is not completely known [18]–[20], [31]. Suggested mechanisms include protein phosphatase-2A (PP2A) activation and inactivation of Akt/ Protein kinase B (PKB), which attenuate the insulin response [18], [31]. Our results of decreased plasma DG and ceramide content due to high dietary intake of n-3 fatty acids in the fatty fish group are consistent with the observations that dietary exposure to EPA and DHA reduce DG and ceramide production in murines [22]. Taken together, these results and the fact that the prevalence of impaired glucose tolerance and type 2 diabetes is lower in populations consuming large amounts of n-3 fatty acids, supports the concept that DG and ceramides may be the link between n-3 fatty acids and insulin resistance.

We also found a decrease in lysoPC in the fatty fish group, which may be related to anti-inflammatory effects of n-3 fatty acids [8]. LysoPC is the major bioactive lipid component of oxidized LDL and may be responsible for many of the inflammatory effects of oxidized LDL [29], [30]. As expected, EPA and DHA were related with the plasma long chain TG as well as EPA bound cholesterol ester and lysoPC. They were also related significantly with the plasmalogens, the endogenous antioxidants [32], which can be explained by a high affinity of EPA and DHA for acylation to the sn-2 position of plasmalogen phospholipids [33].

All subjects were using multiple medications, which may have confounded the possible effects of fish consumption. However, it is not possible to study the selected study population without numerous medications. All subjects were using statins, which are known to cause drug-specific changes in the plasma lipidomic profile [34].

In conclusion, this study indicates that the 8-week consumption of fatty fish decreased plasma lipids which are potential mediators of lipid-induced insulin resistance and inflammation, and may be related to the protective effects of fatty fish on the progression of CHD or insulin resistance. Future studies are needed to find out if these bioactive lipid components are changed due to fish consumption in other tissues, like in skeletal muscle, adipose tissue or immune cells as well.

## Supporting Information

Protocol S1 (in Finnish)
(0.12 MB DOC)

Checklist S1 CONSORT checklist
(0.06 MB DOC)
