# Supplementary material for: Fatty Fish Intake Decreases Lipids Related to Inflammation and Insulin Signaling—A Lipidomics Approach
Source: PLoS One. 2009 Apr 23;4(4):e5258. doi: 10.1371/journal.pone.0005258 (PMC2669180; doi:10.1371/journal.pone.0005258)
Supplement: Protocol S1 — (in Finnish) (0.12 MB DOC) [file pone.0005258.s001.doc]

**Kalan käytön vaikutukset rytmihäiriöherkkyyteen ja sydäntautien vaaratekijöihin sydäninfarktipotilailla**

Tutkimussuunnitelma

Arja Erkkilä, dosentti, FT, Kansanterveystieteen ja yleislääketieteen laitos ja Kliinisen ravitsemustieteen laitos, Kuopion yliopisto

Ursula Schwab, dosentti, FT, Kliinisen ravitsemustieteen laitos ja Elintarvikkeiden terveysvaikutusten tutkimuskeskus, Kuopion yliopisto ja Kliinisen ravitsemuksen yksikkö, Kuopion yliopistollinen sairaala

Seppo Lehto, dosentti, LT, Sisätautien klinikka, Kuopion yliopistollinen sairaala

Hanna Mussalo, LT, Kliininen fysiologia ja isotooppilääketiede, Kuopion yliopistollinen sairaala

Leena Pulkkinen, dosentti, FT, Elintarvikkeiden terveysvaikutusten tutkimuskeskus, Kuopion yliopisto

Matti Uusitupa, professori, Kliinisen ravitsemustieteen laitos ja Elintarvikkeiden terveysvaikutusten tutkimuskeskus, Kuopion yliopisto

ABSTRAKTI

Kalan käyttöön ja pitkäketjuisiin omega-3 rasvahappoihin on liitetty edullisia vaikutuksia sydäntautien vaaratekijöihin ja sydäntautien esiintyvyyteen ja kuolleisuuteen. Kuitenkaan rasvaisen ja vähärasvaisen kalan vaikutusta useisiin vaaratekijöihin esim. rytmihäiriöihin ja insuliiniresistenssiin sekä geenien ilmentymiseen ei ole selvitetty. Aiemmissa, epidemiologissa tutkimuksissamme runsas kalan käyttö on ollut yhteydessä pienempään kuolleisuuteen ja pienempään ateroskleroosin etenemiseen sydänpotilailla. Tavoitteena on tutkia rasvaisen ja vähärasvaisen kalan pitkäaikaisvaikutuksia rytmihäiriöherkkyyteen, sydäntautien vaaratekijöihin (inflammatoriset ja hemostaattiset merkkiaineet, seerumin lipidit, insuliiniherkkyys) ja geenien ilmentymiseen rasvakudoksessa ja leukosyyteissa sydäninfarktipotilailla. Kalojen ryhmittely rasvaisuuden perusteella mahdollistaa kalan rasvan vaikutuksien selvittämisen riippumatta muista kalan ravintoaineista esim. proteiinit. Tutkimus toteutetaan kontrolloituna, satunnaistettuna rinnakkaiskokeena, joka kestää 8 viikkoa. Tutkimukseen rekrytoidaan 45 sydäninfarktin sairastanutta koehenkilöä sairaalan poistoilmoitusrekisteristä. Tutkimuksessa on kolme ruokavaliota: rasvainen kala (4 ateriaa/vko), vähärasvainen kala (4 ateriaa/vko) ja kontrolli, jolloin syödään vähärasvaista lihaa tai kanaa. Tutkimus aloitetaan syksyllä 2005 ja suoritetaan Kliinisen ravitsemustieteen laitoksessa, Kuopion yliopistossa. Yhteistyötahoihin kuuluvat Sisätautien klinikka, KYS, joka vastaa koehenkilöiden etsimisestä poistoilmoitusrekisteristä, Kliinisen fysiologia ja isotooppilääketiede, KYS, joka vastaa rytmihäiriöalttiuden mittauksista ja Elintarvikkeiden terveysvaikutusten tutkimuslaitos, Kuopion yliopistossa, joka vastaa geenien ilmentymiseen liittyvästä analytiikasta.

Tutkimuksen tulokset julkaistaan tieteellisissä julkaisusarjoissa. Sydän- ja verisuonitaudit ovat edelleen tärkein yksittäinen kuolinsyy (35-40% kuolemista Suomessa vuonna 1999). Aiempien tutkimusten perusteella omega-3 rasvahappojen vaikutukset voivat olla jopa verrattavissa sydänlääkkeiden vaikutuksiin. Kuitenkin vain vajaa 40 % suomalaisista raportoi käyttävänsä kalaa vähintään kahdesti viikossa, mikä on Suomen Sydänliiton suositus. Sydänpotilaat voisivat hyötyä tätäkin suuremmasta kalan käytöstä. Tutkimus hyödyttää sydänpotilaita antamalla tarkempaa tietoa ruokavalion keinoista ehkäistä uusia tautitapahtumia. Ravitsemushoito on myös yhteiskunnan kannalta kustannusvaikuttava hoitomuoto.

1. TAUSTA

*Epidemiologiset tutkimukset*

Kalan käytön ja pitkäketjuisten omega-3 rasvahappojen (eikosapentaeeni- ja dokosaheksaeenihappo) saannin yhteyttä sydän- ja verisuonitautien vaaratekijöihin on tutkittu monissa tutkimuksissa (1). Erityisesti pienempi äkkikuoleman vaara on liitetty runsaaseen omega-3 rasvahappojen saantiin ja pitoisuuksiin veressä (2, 3). Tutkimuksessa, jossa lisättiin rasvaisen kalan käyttöä, kokonaiskuolleisuus ja kuolleisuus sydänperäisiin syihin pienenivät miehillä, joilla oli ollut sydäninfarkti (4).

*Mahdolliset mekanismit*

Omega-3 rasvahapot voivat vaikuttaa edullisesti moniin sydäntautien vaaratekijöihin. Omega-3 rasvahapoilla on rytmihäiriöherkkyyttä vähentävää vaikutusta solu- ja eläinmalleissa (5), kuitenkin tämä yhteys on todentamatta kliinisessä kokeessa, jossa lisätään kalan käyttöä. Omega-3 rasvahappojen veren triglyseridipitoisuutta pienentävä vaikutus on todettu lukuisissa tutkimuksissa (6). Omega-3 rasvahapot alentavat verenpainetta, tosin vaikutus on pieni (7). Insuliiniresistenssi on tärkeä sydäntautien vaaratekijä (8). Omega-3 rasvahappojen vaikutus veren glukoosi- ja insuliiniaineenvaihduntaan on vielä epäselvä (9). Kalan käyttö ja omega-3 rasvahapot voivat vaikuttaa edullisesti myös seuraaviin sydäntautien vaaratekijöihin: hemostaattiset tekijät, verihiutaleiden kokkaroituminen, tulpan muodostus ja tulehdusta välittävät sytokiinit. Tutkimustulokset näistä päätemuuttujista ovat vielä kuitenkin ristiriitaisia. Kalan rasvat voivat olla yhteydessä myös masennukseen (10).

*Nutrigenomiikka*

Nutrigenomiikan avulla voidaan tutkia ravintoaineiden vaikutusta geenien ilmentymiseen ja tällä tavoin voidaan löytää molekylaarisia mekanismeja siihen, miksi esim. seerumin lipidi- ja glukoosipitoisuuksissa on eroa eri ruokavalioiden vaikutuksesta (11). Omega-3 rasvahappojen oletetaan vaikuttavan geenien ilmentymiseen rasvakudoksessa, millä voi olla tärkeä merkitys esim. insuliiniresistenssin, hyperlipidemioiden ja lihavuuden kehittymisessä sekä tulehdusta välittävien aineiden tuottoon rasvakudoksessa.

*Kala vs. kalaöljyvalmisteet*

Useimmat kliinisistä kokeista on tehty käyttäen kalaöljyvalmisteita omega-3 rasvahappojen lähteenä. Omega-3 rasvahappojen oletetaankin olevan tärkein tekijä kalan terveysvaikutusten takana. Myös muut ravintoaineet, kuten kalanproteiini, voivat vaikuttaa edullisesti sydäntautien vaaratekijöihin (12). Kala on vierasaineiden, kuten dioksiinien ja elohopean lähde ja tämän takia kalan käyttöön on esitetty liittyvän haittavaikutuksia. Kun arvioidaan kokonaisriskiä, nämä haitat ovat kuitenkin merkitykseltään vähäisempiä kuin kalan edulliset terveysvaikutukset (13). Kalan käyttöön liittyvät riskit pienenevät, kun käytetään eri kalalajeja vaihdellen ja vältetään isojen petokalojen runsasta käyttöä.

*Liittyminen muihin hankkeisiin*

Aiemmassa tutkimuksessamme runsas kalan käyttö ja suuri omega-3 rasvahappojen pitoisuus seerumin lipideissä olivat yhteydessä pienempään kokonaiskuolleisuuteen sydänpotilailla (14). Runsas kalan käyttö oli myös yhteydessä hitaampaan ateroskleroosin etenemiseen postmenopausaalisilla naisilla (15). Aiemmat tutkimusryhmän työt ovat perustuneet epidemiologisiin havaintoihin, joita tämä hanke jatkaa selvittämällä kokeellisessa asetelmassa kalan vaikutuksia sydäntautien vaaratekijöihin. Lisäksi postmenopausaalisten naisten aineistossa (15) on meneillään jatkotutkimus, jossa selvitetään seerumin omega-3 rasvahappojen yhteyttä ateroskleroosin etenemiseen. Tämä tutkimus on pilottitutkimus pitempikestoiselle projektille, johon rekrytoidaan enemmän koehenkilöitä.

2. TAVOITTEET

Tavoitteena on tutkia rasvaisen ja vähärasvaisen kalan vaikutuksia sydän- ja verisuonitautien vaaratekijöihin sydäninfarktipotilailla. Kalojen ryhmittely rasvaisuuden perusteella mahdollistaa kalan rasvan vaikutuksien selvittämisen riippumatta muista kalan ravintoaineista esim. proteiinit. Tutkimus toteutetaan kontrolloidussa, satunnaistetussa rinnakkaisasetelmassa ja se kestää kaksi kuukautta. Päävastemuuttujat ovat inflammatoriset merkkiaineet, rytmihäiriöherkkyys ja geenien ilmentyminen valkosoluissa ja rasvakudoksessa. Erityisesti inflammaation liittyvien geenien ilmentymistä tutkitaan.

3. MERKITYS

Sydän- ja verisuonitaudit ovat edelleen suurin yksittäinen kuolinsyy (35-40% kuolemista Suomessa vuonna 1999). Isossa, sydäninfarktipotilailla toteutetussa tutkimuksessa kalaöljyvalmistetta (1 g/pv) käyttäneellä ryhmällä sydän- ja verisuonitapahtumien vaara oli 11% pienempi, sydänkuolemien vaara 17% pienempi ja äkkikuolemien vaara jopa 26% pienempi kuin kontrolliryhmällä (16). Toisin sanoen vaikutukset voivat olla jopa verrattavissa sydänlääkkeiden vaikutuksiin. Esimerkiksi sepelvaltimotaudin päätetapahtumat vähenevät 24-37% statiinilääkityksellä (17). Omega-3 rasvahappoannos, 1g/pv, jolla on ollut edullisia vaikutuksia ja jota Amerikan sydänyhdistys suosittelee sydänpotilaille (18), on mahdollista saada ruokavaliosta lisäämällä rasvaisen kalan käyttöä (3-4 annosta/vko).

Kotimaisen kalan kulutus filepainona ilmoitettuna oli 5.6 kg/hlö/v, kun tarkastellaan ihmisravinnoksi tarjolla olevaa määrää, ja ulkomaisen kalan kulutus tuotepainona ilmoitettuna oli 9.4 kg/hlö/v vuonna 2003 (Riistan ja kalantutkimus laitos, www.rktl.fi/tilastot). Vain vajaa 40 % suomalaisista raportoi käyttävänsä kalaa vähintään kahdesti viikossa (19), mikä on Suomen Sydänliiton suositus. Sydänpotilaat voisivat hyötyä tätäkin suuremmasta kalan käytöstä. Jos kalan käyttöä lisättäisiin suositellulle tasolle, tämä voisi vähentää sydäntautitapahtumien määrää. Kalankäytön lisäämisellä on myös elinkeinopoliittisia vaikutuksia esim. kalanpyyntiin, -viljelyyn ja –jalostukseen.

4. TUTKIMUSMENETELMÄT

*Ruokavaliot*

Tutkittavat satunnaistetaan yhdelle kolmesta tutkimusruokavaliosta: 1) kontrolli (vähärasvainen liha), 2) vähärasvainen (valkoinen) kala kuten hauki, ahven, seiti ja turska tai 3) rasvainen kala kuten lohi, silakka, siika tai muikku. Kalaruokavalioiden aikana syödään vähintään neljä kala-ateriaa viikossa ja kalan määrä aterialla tulisi olla 100-150 g. Pitkäketjuisten omega-3 rasvahappojen saanti olisi vähärasvaisen kalaruokavalion aikana 0.1-0.2 g/pv ja rasvaisen kalaruokavalion aikana 0.8-1.4 g/pv. Kontrolliruokavaliossa pääruokien raaka-aineena käytetään vähärasvaista lihaa (nauta tai sika) ja kanaa. Kalan käyttö kontrolliruokavalion aikana tulisi olla korkeintaan 1 kala-ateria viikossa. Kaikkien ruokavalioiden aikana muiden kuin omega-3 rasvahappojen saannin osalta tavoitteena on sydänpotilaille suositeltu ruokavalio (8). Ruokavalioiden toteuttamisesta annetaan neuvontaa sekä kirjallista materiaalia. Kalaöljyvalmisteiden käyttö ei ole sallittua tutkimuksen aikana. Liikuntatottumusten ja alkoholin käytön tulisi pysyä samana tutkimuksen ajan.

*Koehenkilöt*

Tutkimus on asetelmaltaan satunnaistettu, kontrolloitu rinnakkaiskoe. Tutkittavat (n=45) rekrytoidaan Kuopion yliopistollisen sairaalan poistoilmoitusrekisterin sydäninfarktipotilaista. Poistoilmoitusrekisteristä identifioiduille henkilöille lähetetään kirje, jossa tiedustellaan halukkuutta osallistua tutkimukseen ja he voivat halutessaan ottaa yhteyttä tutkimusajan varaamiseksi. Tutkimukseen valitaan alle 70-vuotiaista miehiä ja naisia, joilla on ollut sydäninfarkti viimeisen 3-12 kuukauden aikana.

Homogeenisen tutkimusjoukon saamiseksi, koehenkilöillä tulee olla käytössä beetasalpaaja ja heillä tulee olla EKG:lla varmennettu sinus-rytmi. Poissulkukritereeihin kuuluvat muut rytmihäiriölääkkeet kuin beetasalpaajat, eteisflimmeri, rytminsiirtolaite, sepelvaltimoiden ohitusleikkaus tai pallolaajennushoito viimeisen 3 kk:n aikana tai suunnitteilla oleva leikkaus, kehon painoindeksi <18.5 tai >30 kg/m^2^, paasto seerumin triglyseridipitoisuus >3.5 mmol/l, paasto kokonaiskolesterolipitoisuus >8 mmol/l, tyypin 1 tai 2 diabetes, paasto glukoosipitoisuus >7 mmol/l, epänormaali maksan, kilpirauhasen tai munuaisten toiminta, runsas alkoholin käyttö (naisilla >16, miehillä >24 annosta/vko), psyykenlääkkeet, tulehduksellinen suolistosairaus, kalaöljyvalmisteiden käyttö viimeisen 3 kk:n aikana, runsas kalan käyttö (>3 ateriaa/vko) ja kala-allergia. Koehenkilöt jatkavat lääkärinsä määräämien sydänlääkkeiden, mukaan lukien lipidilääkkeet, käyttöä tutkimuksen ajan.

*Mittaukset*

Seulonnassa koehenkilöehdokkailta otetaan verinäyte ja tehdään EKG soveltuvuuden selvittämiseksi. Tutkimuksessa verinäytteitä otetaan 3 kertaa (0, 4 ja 8 vko) ja suoritettavat mittaukset on lueteltu taulukossa. Ruokavalion noudattamista seurataan 2 kertaa käyttäen 7 päivän ruokapäiväkirjaa sekä määrittämällä seerumin lipidien rasvahappokoostumus tutkimuksen alussa ja lopussa (20). Tutkittavien tavanomaista ruokavaliota selvitetään 4 päivän ruokapäiväkirjan avulla ennen intervention alkua. Kyselyiden avulla seurataan liikunnan määrää, alkoholin käyttöä ja tupakointia.

Rytmihäiriöherkkyyttä ja autonomisen hermoston toimintaa mitataan 24 tunnin Holter monitoroinnin avulla. Tiedot käsitellään käyttäen WinCPR ohjelmistoa. Analysoitavia suureita ovat ennenaikaiset kammiolyönnit, rytmihäiriöt, sydämen sykkeen vaihtelu, QT aika. Vasemman kammion toiminta arvioidaan sydämen ultraäänitutkimuksella (Acuson) tutkimuksen alussa. Insuliiniherkkyyttä mitataan oraalisen sokerirasituskokeen avulla. Glukoosi- ja insuliinipitoisuuksista lasketaan insuliiniherkkyysindeksi. Koehenkilöiltä otetaan näyte mahan alueen ihonalaisesta rasvakudoksesta tutkimuksen alussa ja lopussa. Seerumin lipidien ja glukoosin aineenvaihduntaan sekä hemostaattisiin ja tulehduksellisiin tekijöihin liittyvien geenien ilmentymistä tutkitaan rasvakudosnäytteestä ja leukosyyteistä nutrigenomiikan menetelmin osalla koehenkilöistä (n=10+10) tutkimusten suurten kustannusten takia. Tutkimuksissa hyödynnetään Affymetrix U133 human slide set (A and B) ja kvantitatiivista PCR:ä.

Taulukko. Suoritettavat mittaukset ja tutkimukset

|  | 0 kk | 4 vko | 8 vko |
| --- | --- | --- | --- |
| *Seulonta:* |  |  |  |
| S-gamma-glutamyylitransferaasi | X |  | (x) |
| S-kreatiniini | X |  |  |
| S-TSH | X |  |  |
| Paino, pituus, vyötärö, lantio | X |  |  |
| Lepo-EKG | X |  |  |
| *Tutkimuksen aikana:* |  |  |  |
| Pieni verenkuva + trombosyytit | X |  | X |
| Paino, vyötärö, lantio, verenpaine | X | X | X |
| Kysely elintavoista (liikunta, alkoholi) | X |  | X |
| 7-päivän ruokapäiväkirja |  | X | X |
| S-lipidien rasvahappokoostumus | X |  | X |
| S-kokonais- ja HDL kolesteroli, triglyseridit | X | X | X |
| Apolipoproteiinit A-1, B | X |  | X |
| Oraalinen glukoosirasitus | X |  | X |
| Sensitiivinen C-reaktiivinen proteiini (hs-CRP) | X |  | X |
| PAI-1 (hemostaattinen markkeri) | X |  | X |
| Fibrinogeeni | X |  | X |
| Kalium, kalsium | X |  | X |
| *Rytmihäiriöherkkyys:* |  |  |  |
| Holter, 24 t monitorointi | X |  | X |
| Sydämen ultraääni (Vas. kammion funktio) | X |  |  |
| *Geenien ilmentyminen:* |  |  |  |
| Rasvakudos ja valkosolu näytteet | X |  | X |
| Affymetrix (n=10+10) | X |  | X |
| Kvantitatiivinen PCR | X |  | X |

*Eettiset kysymykset*

Tutkimussuunnitelma hyväksytetään Pohjois-Savon sairaanhoitopiirin ja Kuopion yliopiston tutkimuseettisellä toimikunnalla. Tutkimus kuvataan tutkittavalle sekä kirjallisesti että suullisesti, jolloin tutkittavalla on mahdollisuus esittää tarkentavia kysymyksiä. Tutkittavilta pyydetään kirjallinen lupa. Tutkittava allekirjoittaa suostumuslomakkeen kahtena kappaleena, joista toinen jää tutkittavalle ja toinen tutkijalle. Tutkittavat voivat keskeyttää tutkimuksen halutessaan. Tutkittaville annetaan tehtyjen mittausten tulokset tutkimuksen päätyttyä lukuun ottamatta perintötekijöiden ilmentymiseen liittyviä tuloksia. Jos laboratoriomittauksissa tai muissa tutkimuksissa löytyy poikkeavia tuloksia, koehenkilöt ohjataan jatkotutkimuksiin.

*Aikataulu*

Koehenkilöiden rekrytointi tehdään syksyllä 2005. Ruokavalioneuvontaa annetaan tutkimuksen alussa ja verinäytteitä otetaan 0, 4 ja 8 viikon kohdalla. Tutkimuksen kliininen, käytännön vaihe loppuu alkuvuodesta 2006.

5. SUORITUSPAIKKA

Hanke toteutetaan pääasiallisesti Kliinisen ravitsemustieteen laitoksessa, Kuopion yliopistossa (Erkkilä, Schwab, Uusitupa). Laitoksen vastuulla ovat tutkimuksen suunnittelu, tulosten analysointi ja raportointi sekä ravitsemusneuvonta, oraaliset glukoosirasitukset, verinäytteiden otto ja peruslaboratoriomääritykset. Koehenkilöiden rekrytointi poistoilmoitusrekisteritietoihin perustuen tehdään yhteistyössä Sisätautien klinikan, Kuopion yliopistollinen sairaala (Lehto) kanssa. Rytmihäiriöherkkyyden mittaukset mukaan lukien Holter monitorointi tehdään yhteistyössä Kliinisen fysiologian ja isotooppilääketieteen osaston kanssa (Mussalo). Geenien ilmentymiseen liittyvistä analyyseistä vastaa Elintarvikkeiden terveysvaikutusten tutkimuskeskus (Pulkkinen) ja ne tehdään yhteistyössä A.I. Virtanen Instituutin kanssa (Seppo Ylä-Herttuala).

Tutkimuksen suorituspaikoissa on tarvittava varustus ja henkilökunta tutkimuksen suorittamiseen.

6. TULOSTEN JULKAISU- JA HYÖDYNTÄMISSUUNNITELMA

Tulokset tullaan julkaisemaan tieteellisissä julkaisusarjoissa. Tutkimus antaa tärkeää uutta tietoa siitä, miten runsas kalan käyttö vaikuttaa sydäntautien vaaratekijöihin sydäninfarktipotilailla. Erityisesti saadaan tietoa kalan rasvan ja proteiinien terveysvaikutuksista. Näitä tietoja voidaan käyttää hyväksi sydänpotilaiden ruokavalio-ohjeiden tarkentamisessa. Koska sydän- ja verisuonitaudit ovat edelleen tärkein yksittäinen kuolinsyy ja sepelvaltimotauti on yksi merkittävimmistä kansantaudeista, ravitsemushoito on tärkeää sekä yksilön terveyden että terveydenhuollon kustannusten kannalta. Lisääntyneellä kalan käytöllä on myös vaikutusta suomalaiseen kalatalouteen. Tarkempi tieto kalaan liittyvistä edullisista terveysvaikutuksista voisi lisätä kuluttajien kiinnostusta ja kalan käyttöä.

8. HANKKEEN BUDJETTI JA RAHOITUSSUUNNITELMA

Hankkeen budjetti on 38 000 e, mihin sisältyy tutkimushoitajan palkkaus 4 kk, 10 000 e, laboratoriotarvikkeita ja määrityksiä 26 200 e sekä elintarvikekuluja 1800 e. Rahoitusta on saatu seuraavilta säätiöiltä: Yrjö Jahnsson, Juho Vainio, Suomen Kulttuurirahasto, Suomen kulttuurirahaston Pohjois-Savon rahasto. Lisäksi geenien ilmentymisen tutkimiseen haetaan lisärahoitusta.

Tutkittaville maksetaan korvaus lisääntyneen kalankäytön aiheuttamista kustannuksista. Sama korvaus maksetaan myös verrokkiryhmään kuuluneille tutkittaville.

Laajempaan tutkimukseen haetaan rahoitusta Suomen Akatemialta, Sydäntutkimussäätiöltä ja Maa- ja Metsätalousministeriöltä.

VIITTEET

1. Balk E, Chung M, Lichtenstein AH, et al. Effects of Omega-3 Fatty Acids on Cardiovascular Risk Factors and Intermediate Markers of Cardiovascular Disease. (Prepared by Tufts-New England Medical Center Evidence-based Practice Center under Contract No. 290-02-0022). Rockville, MD, USA: Agency for Healthcare Research and Quality, 2004.

2. Marchioli R, Barzi F, Bomba E, et al. Early protection against sudden death by n-3 polyunsaturated fatty acids after myocardial infarction: Time-course analysis of the results of the Gruppo Italiano per lo studio della sopravvivenza nell'infarto miocardido (GISSI)-prevenzione. Circulation 2002;105:1897-1903.

3. Albert CM, Campos H, Stampfer MJ, et al. Blood levels of long-chain n-3 fatty acids and the risk of sudden death. N Engl J Med 2002;346:1113-1118.

4. Burr ML, Fehily AM, Gilbert JF, et al. Effects of changes in fat, fish, and fibre intakes on death and myocardial reinfarction: Diet and reinfarction trial (DART). Lancet 1989;2:757-761.

5. Leaf A, Kang JX, Xiao YF, Billman GE. Clinical prevention of sudden cardiac death by n-3 polyunsaturated fatty acids and mechanism of prevention of arrhythmias by n-3 fish oils. Circulation 2003;107:2646-2652.

6. Harris WS. n-3 fatty acids and serum lipoproteins: human studies. Am J Clin Nutr 1997;65 (suppl):1645S-1654S.

7. Geleijnse JM, Giltay EJ, Grobbee DE, Donders AR, Kok FJ. Blood pressure response to fish oil supplementation: metaregression analysis of randomized trials. J Hypertens 2002;20:1493-1499.

8. National Cholesterol Education Program (NCEP) Expert Panel. Detection, Evaluation, and Treatment of High Blood Cholesterol in Adults (Adult Treatment Panel III) final report. Circulation 2002;106:3143-3421.

9. Sirtori CR, Crepaldi G, Manzato E, et al. One-year treatment with ethyl esters of n-3 fatty acids in patients with hypertriglyceridemia and glucose intolerance. Reduced triglyceridemia, total cholesterol and increased HDL-C without glycemic alterations. Atherosclerosis 1998;137:419-427.

10. Peet M, Horrobin DF. A dose-ranging study of the effects of ethyl-eicosapentaenoate in patients with ongoing depression despite apparently adequate treatment with standard drugs. Arch Gen Psychiatry 2002;59:913-919.

11. Pegorier JP, Le May C, Girard J. Control of gene expression by fatty acids. J Nutr 2004;134:2444S-2449S.

12. Demonty I, Deshaies Y, Lamarche B, Jacques H. Cod protein lowers the hepatic triglyceride secretion rate in rats. J Nutr 2003;133:1398-1402.

13. Tuomisto JT, Tuomisto J, Tainio M, et al. Risk-benefit analysis of eating farmed salmon. Science 2004;305:476-7.

14. Erkkilä AT, Lehto S, Pyörälä K, Uusitupa MIJ. n-3 Fatty acids and 5-y risks of death and cardiovascular disease events in patients with coronary artery disease. Am J Clin Nutr 2003;78:65-71.

15. Erkkilä AT, Lichtenstein AH, Mozaffarian D, Herrington DM. Fish intake is associated with reduced progression of coronary artery atherosclerosis in postmenopausal women with coronary artery disease. Am J Clin Nutr 2004;80:626-632.

16. GISSI-Prevenzione investigators. Dietary supplementation with n-3 polyunsaturated fatty acids and vitamin E after myocardial infarction: results of the GISSI-Prevenzione trial. Lancet 1999;354:447-455.

17. Suomen Sydäntautilääkärien yhdistys ry:n asettama työryhmä. Dyslipidemiat. Käypä hoito -suositus. Duodecim 2004;120:1794-1816.

18. Kris-Etherton PM, Harris WS, Appel LJ. Fish consumption, fish oil, omega-3 fatty acids, and cardiovascular disease. Circulation 2002;106:2747-2757.

19. Similä M, Fagt S, Vaask S, et al. The NORBAGREEN 2002 study - Consumption of vegetables, potatoes, fruit, bread and fish in the Baltic countries. In: Nordic Council of Ministers, ed. TemaNord, 2003.

20. Ågren JJ, Julkunen A, Penttilä I. Rapid separation of serum lipids for fatty acid analysis by a single aminopropyl column. J Lipid Res 1992;33:1871-1876.
